# The effect of exercise on the severity of the fatigue in colorectal cancer patients who received chemotherapy in Ahwaz

**Published:** 2010

**Authors:** Abdolali Shariati, Shayesteh Haghighi, Seddigheh Fayyazi, Hamed Tabesh, Mehrnaz Moradi Kalboland

**Affiliations:** * Nursing, Faculty Member, School of Nursing and Midwifery, Ahwaz University of Medical Sciences, Ahwaz, Iran; ** Statistics, Faculty Member, School of Health, Ahwaz University of Medical Sciences, Ahwaz, Iran

**Keywords:** Exercise, fatigue, cancer, chemotherapy

## Abstract

**BACKGROUND:**

One of the common side effects of cancer is fatigue that affects patients’ life quality and leads to disability. Exercise has an important role in improving these patients’ life quality and can be used as a complementary treatment. Moreover, there are few studies on the impact of exercise on fatigue among patients with colon cancer. Therefore, the present study investigated the effects of exercise on the severity of fatigue in patients with colorectal cancer who underwent chemotherapy in Ahwaz.

**METHODS:**

In a quasi-experimental study, the adults with colorectal cancer were enrolled. The sample included 36 people. The study environment included adult hematology and chemotherapy wards of Shefa Hospital in Ahwaz. Data were collected using a demographic form and a questionnaire in order to measure the severity of fatigue. Then, the patients had 40 minutes of exercise, 3 times a week for 4 weeks. The effect of exercise versus fatigue intensity was measured at the end of every week. Data were analyzed using SPSS software.

**RESULTS:**

The mean of the fatigue severity in the weeks after exercise was significantly different from the week before it. Friedman test showed significant differences between all the weeks before and after the exercise. The mean of the fatigue severity was 3.69 on the week 0 (before the exercise), and decreased to 3.57 on the first week after exercise, 3.46 on the second week, 2.58 on the third week, and 1.69 on the forth week.

**CONCLUSIONS:**

Considering the results of this study, exercise and work-out can be an effective factor in reducing fatigue in patients.

In Iran, the incidence of colorectal cancer is about 6 to 7.9 in every 100,000 person each year and it is the fourth prevalent cancer.^1^ The mortality rate in Iran is about 1.198 per 100,000, including 13% of deaths due to gastrointestinal cancers and 5.3% of deaths due to causes other than accidents.^2^

One of the most complicated and commonest problems of the cancer is fatigue. About 72 to 99% of cancer patients suffer from fatigue, which may cause from the process of disease and treatments called cancer-related fatigue.^3^ Cancer-related fatigue is a disabling and continuous phenomenon in patients during and after treatment^4^ can continue for many years after treatment and have negative impact on the life quality.^5^

Some studies reported that fatigue in cancer patients have more negative effects on life quality than other symptoms such as vomit, nausea, pain, and depression.^6^

Stone believed that various complications would emerge in patients’ life following cancer treatment and fatigue is one of them. In a cross-sectional study on 1307 cancer patients in three different medical centers during a period of 30 days using a questionnaire, the effects of fatigue was found to cause problems for 58% of the patients, pain for 22% and vomit and nausea for 18% of the patients. Fifty two percent never complained about fatigue to their physician and only 14% of the patients received medical advices to treat their fatigue. The other significant point is that cancer patients do not receive any education to be prepared for experiencing fatigue.^7^

Patients usually find various ways to fight against fatigue. These methods can include changing sleeping pattern, physical activity, social interaction with friends and family, correction of anemia and some other treatments.^5^

A well-known strategy to reduce fatigue is encouraging patients to have frequent breaks between their daily activities or reduce the level of their activity. However, this strategy, especially practiced on long term can lead to more disabilities on patients. Physical activity helps keeping the balance of energy while lack of activity or too much rest can increase the feeling of fatigue. The more people reduce their activities, the more they feel tired and have less ability to do their daily activities.^5^

All societies use exercise to prevent diseases, improve health and feeling lively. In Safavi study on application and effectiveness of methods used to decrease fatigue in cancer patients undergoing chemotherapy, it was found that the most common used method by patients was lying down and the least used was exercise.^8^

Hewitt et al also found that cancer patients are as twice at risk of motion limitation as others and if they have chronic disease, this risk is five times more.^9^

Exercise and mild activity can increase good feeling and activity tolerance in people^6^ and affect not only the body but also emotional and psychological status of the people.^10^

In a study by Corner et al, the effects of exercise on physical ability, feeling good and life quality of patients with tumor and blood cancer were investigated and the results showed that following intervention, total outcome including physical ability, physical activity, pain reduction, and fatigue were significantly increased.^11^

Also, random clinical trials on cancer patients who were between 21 and 65 years old showed that physical activity reduced cancerrelated fatigue during and after the treatment.^12^

The important point is that in spite of the high prevalence of cancer-related fatigue and the existence of strategies to reduce this problem, fatigue in colorectal cancer patients was ignored.^13^ Most studies on the topic have focused on the effect of exercise on patients with breast cancer. Therefore, this study was conducted to find the effect of exercise on the severity of fatigue in patients with colorectal cancer who underwent chemotherapy in Shefa Hospital of Ahwaz in 2005. We hoped that the results of this study could be an effective step towards reducing this problem.

## Methods

In this quasi-experimental study, adult patients with colorectal cancer referred to Shefa Hospital of Ahwaz in 2005 were included. The sample size was 36 and the researcher presented in the research environment during the working hours to select the hospitalized patients who had the entry criteria and were willing to participate in the study. The entry criteria included having colorectal cancer, under-going chemotherapy with special protocol, being 18 to 65 years old, being diagnosed with colorectal cancer at least 3 months ago and being able to do exercise.

Patients with heart diseases, diabetes mellitus, hypertension, anemia, decreased white blood cells, asthma, imbalance, severe pain, and bone metastases were excluded from the study. Selection and exclusion of the patients were based on their medical files and approval of their physicians.

At the beginning, after explaining the effects of exercise on body and the method and receiving permission from the patients, each of them completed a questionnaire including demographic data and a fatigue assessment form to determine the severity of their primary fatigue. Demographic and disease data questionnaire included 21 questions about height, weight, age, gender, education, job, income, disease level, disease duration, treatments received so far, type and amount of medications in chemotherapy, number of chemotherapy periods, other medications, other diseases such as diabetes mellitus, heart diseases etc., their previous exercises before and during the disease, and the type of exercise and its amount. The fatigue assessment form also included 10 questions assessing current fatigue, usual fatigue in past 24 hours and the most severe fatigue that patients had experienced in past 24 hours, as well as the effect of fatigue on their general activity, mood, walking ability, communicating with others, and enjoying life in past 24 hours. Each of these questions was scored based on a scale of 0 to 10. Reliability of this instrument was approved with 98% correction coefficient in a study in Canada by Groenwald.^14^

Also, in the study of Rad in 2001, the correlation coefficient was found to be as r = 85.^15^

The exercise included the soft motions of the body organs and head and neck for 40 minutes. Before starting the exercise, the stage of warming body was practiced for 5 minutes and then flexion and extension of the head and neck and extremity joints was performed for 30 minutes. At the end stage, body cooling was carried out for 5 minutes. Exercise was done 3 times a week for a month between the chemotherapy sessions. The patients participating in the study were divided into 3 groups of 11 to 13 people, one group for women and two other groups for men. Exercises were done by a physical trainer working at Shahid Chamran University of Ahwaz. It was hold in the gym of the Medical University.

Right before the exercise, the patients’ blood pressure and pulse were controlled as the basic vital signs and after the exercise also, their blood pressure and pulse was recorded so that in case of any problem, they would be excluded from the study. The questionnaire on fatigue was recorded at the time of sample selection, after starting exercise, at the end of each week and finally at the end of the exercise program to assess their fatigue.

The data which collected by the questionnaires were analyzed using Friedman test via SPSS version 13 software.

## Results

The patients in the study consisted of 69% men and 31% women. Of all, 77.8% had no exercise before the disease and 47.2% had often fatigue. Twelve patients (33.3%) were in the grade 3 of the disease and 9 (25%) were diagnosed 6 months ago.

The findings of the study showed that the mean of fatigue severity during the exercise weeks were different from the week before the exercise. The mean of the fatigue decreased from week 0 (before the exercise) to week 4 during the exercise. Friedman test showed a significant difference between all weeks from the first to the fourth (p < 0.01) (Table 1).

**Table 1:** The mean of the fatigue severity at the weeks of exercise

Comparing weeks	Week 0 (before the exercise) Fatigue Severity = 3.69	First week of exercise Fatigue Severity = 3.57	Second week of exercise Fatigue Severity = 3.46	Third week of exercise Fatigue Severity = 2.58
The third week of exercise Fatigue Severity = 2.58	p < 0.01 Z = -3.173	p < 0.01 Z = -3.481	p < 0.01 Z = -4.02/4	---
The third week of exercise Fatigue Severity = 1.69	p < 0.01 Z = -3.370	p < 0.01 Z = -3.949	p < 0.01 Z = -4.481	p = 0.002 Z = -3.170

## Discussion

According to the findings of this study, most subjects had not exercised before their disease. Exercising increases the physical ability and tolerance by improving cardiovascular and musculoskeletal conditions. Those who do not exercise show less strength against the diseases and their complications.^16^ Also, almost half of the patients in this study had sometimes felt fatigue before the intervention. Fatigue in patients suffering from cancer is a prevalent and annoying symptom during the disease or its treatment.^11^

One third of the subjects in the study were in grade 3 of their disease and the rest were in grades 1 and 2. Six months were passed from the disease diagnosis of 25% of the patients. Various studies showed that the more time passed from the disease, the more patients would feel fatigue.^3^,^17^

According to the objective of this study, comparing the severity of fatigue before and after an exercising program, the findings showed that the mean of the fatigue on the third and fourth weeks were significantly different from the beginning and the first and second weeks; also the fourth week was significantly different from the third week. Dimeo et al found that aerobic exercise program improves physical activity of the patients during the recovery from tumor surgery.^18^ Also, Windsor et al revealed that mild walking improves physical condition of patients undergoing physiotherapy without significant increase in their fatigue; the improvement of the physical condition is necessary to recover from fatigue due to radiotherapy.^19^

The results showed that exercising reduced fatigue in patients. Dimeo et al also said that a daily exercising program in accordance with the individual tolerance can reduce lack of physical functioning related to the treatments in patients with blood malignancy undergoing chemotherapy.^20^ The present study can help nurses to solve one of the main problems of their patients by providing a method to prevent and control fatigue in colorectal cancer patients. Also, exercise as a care program can be a great help to nursing management by reducing treatment costs.

Considering the conducted studies and experiences of the researchers during this study, it is recommended that clinical nurses working in oncology wards receive explanations about the necessity of using exercising programs so that they include such programs in their caring activities for their patients and by giving pamphlets to the patients, in order to perform the exercises.

The authors declare no conflict of interest in this study.
